# Sigma-1 Agonist Binding in the Aging Rat Brain: a MicroPET Study with [^11^C]SA4503

**DOI:** 10.1007/s11307-015-0917-6

**Published:** 2015-12-04

**Authors:** Nisha K. Ramakrishnan, Anniek K. D. Visser, Anna A. Rybczynska, Csaba J. Nyakas, Paul G. M. Luiten, Chantal Kwizera, Jurgen W. A. Sijbesma, Philip H. Elsinga, Kiichi Ishiwata, Rudi A. J. O. Dierckx, Aren van Waarde

**Affiliations:** Department of Nuclear Medicine and Molecular Imaging, University Medical Center Groningen, University of Groningen, Hanzeplein 1, 9713 GZ Groningen, The Netherlands; Division of Imaging Sciences and Biomedical Engineering, King’s College London, Strand, London, WC2R 2LS UK; Research Group of Molecular Neurobiology, University of Groningen, Nijenborgh 7, 9747 AG Groningen, The Netherlands; Department of Morphology and Physiology, Semmelweis University, 17 Vas, H-1088 Budapest, Hungary; Southern Tohoku Research Institute for Neuroscience, 7-115 Yatsuyamada, Koriyama, 963-8052 Japan

**Keywords:** Senescence, Sigma-1 receptor, Agonist binding, Positron emission tomography, Kinetic modeling, Brainstem, Hypothalamus

## Abstract

**Purpose:**

Sigma-1 receptor ligands modulate the release of several neurotransmitters and intracellular calcium signaling. We examined the binding of a radiolabeled sigma-1 agonist in the aging rat brain with positron emission tomography (PET).

**Procedures:**

Time-dependent uptake of [^11^C]SA4503 was measured in the brain of young (1.5 to 3 months) and aged (18 to 32 months) Wistar Hannover rats, and tracer-kinetic models were fitted to this data, using metabolite-corrected plasma radioactivity as input function.

**Results:**

In aged animals, the injected probe was less rapidly metabolized and cleared. Logan graphical analysis and a 2-tissue compartment model (2-TCM) fit indicated changes of total distribution volume (*V*_T_) and binding potential (*BP*_ND_) of the tracer. *BP*_ND_ was reduced particularly in the (hypo)thalamus, pons, and medulla.

**Conclusions:**

Some areas showed reductions of ligand binding with aging whereas binding in other areas (cortex) was not significantly affected.

**Electronic supplementary material:**

The online version of this article (doi:10.1007/s11307-015-0917-6) contains supplementary material, which is available to authorized users.

## Introduction

Sigma-1 receptors are widely distributed throughout the brain and are expressed mainly on neurons, with the highest expression levels in various cranial nerve nuclei in the midbrain, pons, and medulla [1–4]. These receptors are ligand-regulated molecular chaperones in the endoplasmatic reticulum and play a modulatory role in intracellular calcium signaling [5]. Activated sigma-1 receptors translocate from the endoplasmatic reticulum to the plasma membrane and modulate the activity of ion channels and neurotransmitter release [5]. Sigma-1 receptor stimulation can increase the extracellular concentrations of dopamine [6–8], acetylcholine [9, 10], and glutamate [11–13]. Sigma-1 receptors have been implied in cellular differentiation [14], neuritogenesis [15], neuroprotection, and cognition [16].

Aging has a strong impact on neurotransmission in the mammalian brain. Particularly, the dopaminergic system has been widely examined, not only with *in vitro* techniques but also with *in vivo* imaging. Substantial losses of dopamine D_1_ and D_2_ receptors binding with increasing age have been observed in micro-positron emission tomography (microPET) studies of young (2 to 6 months) and old (18 to 24 months) rats [17, 18]. Cholinergic deficits have also been detected in this animal species, using microPET and the presynaptic cholinergic marker [^18^F]fluoroethoxybenzovesamicol. [19]

In contrast to the extensive data on dopaminergic and cholinergic neurotransmission, sigma-1 receptors in the aging rodent brain have been little examined. Imaging studies in rats have not yet been reported, and published data from *in vitro* assays concerned either the whole brain [20–23] or only a single brain region, i.e., the striatum [24]. Moreover, most of these data were acquired with sigma ligands which lacked subtype-selectivity [20, 21, 24]. For this reason, we performed quantitative microPET studies with a subtype-selective radioligand in living rats of different ages and determined sigma-1 receptor binding potentials throughout the brain. We were interested in answering the question whether sigma-1 receptor populations in various brain regions are differently affected by healthy aging and whether observed changes (if any) could be related to changes of rodent physiology at advancing age.

## Materials and Methods

### Radioligand

The ligand 1-[2-(3,4-dimethoxyphenethyl)]-4-(3-phenylpropyl)piperazine ([^11^C]SA4503) was prepared by reaction of [^11^C]methyl iodide with 4-O-demethyl SA4503, according to a published method [25]. The decay-corrected radiochemical yield was ~24 %, the specific radioactivity was >100 TBq/mmol at the moment of injection, and radiochemical purity was >98 %. The [^11^C]SA4503 solution had a pH of 6.0 to 7.0.

### Animals

Male Wistar Hannover rats were obtained from Harlan (The Netherlands) and Semmelweis University Budapest. Animals from the following age groups were included: 1.5 (*n* = 9), 3 (*n* = 10), 18 (*n* = 5), 24 (*n* = 4), and 32 (*n* = 5) months. About 40 % of the rats aged 18 to 32 months originally intended for this study were found to have pituitary tumors which affected tracer kinetics in the brain [26]. The microPET data of these animals were thus not included in the current manuscript. Since any differences between the 1.5 and 3 month groups, or the 18, 24, and 32 month groups were small and in most cases not statistically significant, we decided to pool data for young (1.5 to 3 months) and aged (18 to 32 months) rats in the following Results and Discussion sections.

The rats were housed in Makrolon cages on a layer of wood shavings at 21 ± 2 °C and a fixed 12-h light–dark regime (lights on at 7:00 a.m.). Food (standard laboratory chow, RMH-B, Hope Farms, The Netherlands) and water were available *ad libitum*. After arrival, the rats were allowed to acclimatize for at least seven days. The study protocol was approved by the Institutional Animal Care and Use Committee of the University of Groningen. All experiments were performed by licensed investigators in compliance with the law on animal experiments of The Netherlands. Important data concerning the animals (purpose of use, body and brain weights, injected tracer dose, and mass) are provided in Table 1.

**Table 1:** Animal data

	Young (1.5 to 3 months)	Aged (18 to 32 months)
Scanned with rapid arterial blood sampling (*n*)	12	10
Metabolite analysis (*n*)	7	8 (4 were also scanned)
Brain TACs used for kinetic analysis (*n*)	12	14
Body weight (g)	215 ± 3 (1.5 months, *n* = 9)	605 ± 28
	308 ± 8 (3 months, *n* = 10)	
Brain weight (g)	1.73 ± 0.03 (1.5 months)	2.15 ± 0.08
	1.90 ± 0.04 (3 months)	
Injected tracer dose (MBq)	14.7 ± 2.8	26.8 ± 6.2
Injected mass (nmol, max.)	0.15 ± 0.03	0.27 ± 0.06
Injected mass (pmol/g, max.)	5.7 ± 1.1	4.5 ± 1.0

### Arterial Blood Sampling

Before microPET scanning, rats were anesthetized with isoflurane in medical air (5 % for induction and 2 % for maintenance). An incision was made parallel to the femoral artery. The femoral artery was separated from the femoral vein and temporarily ligated to prevent leakage of blood. A small incision was made in the artery, and a cannula was inserted (0.8-mm outer, 0.4-mm inner diameter). The cannula was secured to the artery with a suture and attached to a syringe filled with heparinized saline.

From each rat, fifteen arterial blood samples (volume 0.1 to 0.15 ml) were drawn at 0.25, 0.5, 0.75, 1, 1.25, 1.5, 2.0, 3, 5, 7.5, 10, 15, 30, 60, and 90 min after [^11^C]SA4503 injection. Plasma was obtained from these blood samples by centrifugation (5 min in Eppendorf-type centrifuge at 13,000 × g). Radioactivity in plasma samples (25 μl) was determined using a calibrated gamma counter (CompuGamma CS 1282, LKB-Wallac, Turku, Finland)

In separate groups of rats, larger volumes of blood ranging from 0.4 to 1.6 ml were collected at 5, 10, 20, 40, and 60 min, and a metabolite analysis was performed using a published method [27]. Briefly, plasma was obtained by centrifugation (2 min in Eppendorf-type centrifuge at 13,000 × g) and de-proteinized using one third the volume of 20 % trichloroacetic acid in acetonitrile. The mixture was centrifuged for 2 min at 13,000 × g, and the supernatant was injected in a reversed-phase HPLC system to separate the parent tracer from its metabolites (μBondapak C18 column, 7.8 × 300 mm, mobile phase acetonitrile/50 mM sodium acetate pH 7.2, 1/1, v/v, flow rate 3 ml/min). The eluate was collected in 30-s fractions for 15 min, and radioactivity in the samples was counted. The results were expressed as the percentage of total plasma radioactivity representing parent tracer.

### Scanning

Two rats were scanned simultaneously in each scan session, using a Siemens/Concorde microPET camera (Focus 220). They were placed in the camera in transaxial position with their heads and neck in the field of view. Body temperature of the animals was maintained with heating mats and electronic temperature controllers. Circulation and respiration could be monitored with a BioVet system (M2M Imaging, Cleveland, OH). First, a transmission scan of 515 s with a Co-57 point source was obtained for attenuation and scatter correction of 511 keV photons by tissue. Subsequently, the first rat was injected through the penile vein with [^11^C]SA4503 (31 ± 16 MBq, volume <1 ml). This dose of [^11^C]SA4503 results in maximally 5.0 to 7.5 % sigma-1 receptor occupancy throughout the rat brain, whereas nonspecific binding of the tracer is between 19 and 25 % of total uptake of radioactivity in all the studied brain regions [28, 29]. The emission scan was started with tracer injection of the first rat, whereas the second rat was injected a few minutes later. A list-mode protocol was used with 90-min acquisition time (analysis performed for the first 74 min from tracer injection). The list-mode data of the emission scans were split according to position on the Y-axis and were reframed for each rat independently, into a dynamic sequence of 8 × 30 s, 3 × 60 s, 2 × 120 s, 2 × 180 s, 3 × 300 s, 3 × 600 s, 1 × 720 s, and 1 × 960 s frames relative to the animal’s injection time. The data were reconstructed per time frame employing an iterative reconstruction algorithm (ordered subsets expectation maximization, OSEM 2D with Fourier rebinning, four iterations, and 16 subsets). The final datasets consisted of 95 slices with a slice thickness of 0.8 mm and an in-plane image matrix of 128 × 128 pixels. Voxel size was 0.5 × 0.5 × 0.8 mm. The linear resolution at the center of the field-of-view was about 1.5 mm. Data sets were fully corrected for decay, random coincidences, scatter, and attenuation.

### Data Analysis

Using Inveon Research Workplace (Siemens), three-dimensional regions of interest (ROIs) were drawn on an MRI template of the rat brain [30], both over the whole brain and individual brain regions (bulbus, cortex, striatum, thalamus, hypothalamus, amygdala, midbrain, pons + medulla, and cerebellum). PET images were co-registered with this MRI template, and the regions of interest were transferred from MRI to PET. Time-activity curves (TACs) were obtained for each of these regions. The results were expressed as dimensionless standardized uptake values (SUVs): [tissue activity concentration (MBq/g) × body weight (g) / injected dose (MBq)], assuming a specific gravity of 1 g/ml for brain tissue and blood plasma.

Kinetic analysis was performed by fitting a two-tissue compartment model (2-TCM) to the dynamic PET data using metabolite-corrected arterial plasma radioactivity as input function. Uncorrected whole blood radioactivity was used to estimate the contribution of radioactivity in blood to the measured brain radioactivity. The plasma TAC of each animal was corrected for metabolites using an exponential function obtained from the average metabolite curve of the metabolite analysis rats from the same age group. Software routines for MatLab 7 (The MathWorks, Natick, MA), written by Antoon T.M. Willemsen (University Medical Center Groningen), were used for curve fitting. The cerebral blood volume was fixed at 3.6 % [31], and the rate constants *K*_1_ (from arterial plasma to tissue), *k*_2_ (from tissue to arterial plasma)_,_*k*_3_ (from free to bound compartment in tissue), and *k*_4_ (from bound to free compartment in tissue) were estimated from the curve fit. Non-displaceable volume of distribution (*V*_ND_) was calculated as *K*_1_/*k*_2_, non-displaceable binding potential (*BP*_ND_) as *k*_3_/*k*_4_, and total distribution volume (*V*_T_) as *K*_1_/*k*_2_ * (1 + *k*_3_/*k*_4_).

In kinetic modeling for brain regions, *V*_ND_ was fixed to the estimated value for the whole brain of each individual rat. Additionally, Logan graphical analysis [32] was used to obtain cerebral distribution volume (*V*_Logan_). The Logan fit was started at 20 min, and the parameter for cerebral blood volume was again fixed at 3.6 % [31]. *V*_Logan_ of the tracer was estimated from the curve fit.

### Biodistribution

After the scanning period, the animals were terminated under deep anesthesia by extirpation of the heart. Blood was collected, and plasma and a cell fraction were obtained from the blood sample by short centrifugation (10 min at 13,000 × g). Several tissues were excised and weighed. Radioactivity in tissue samples and in a sample of the injected tracer solution (infusate) was measured using a gamma counter with automatic decay correction. The results were expressed both as SUV and as a ratio (SUV tissue/SUV plasma).

### Statistics

All results are expressed as mean ± SEM. Differences between groups were examined using *t* test (for area under the curves and whole brain 2-TCM parameters) and 2-way ANOVA (biodistribution, brain and periphery analyzed separately and regional 2-TCM data), followed by a post hoc Bonferroni test, where applicable. A *P* value <0.05 was considered statistically significant. Correlations were assessed using Pearson correlation coefficient (*r*), and considered strong when *r*^2^ was at least 0.7.

## Results

### PET Images

**Fig. 1:**
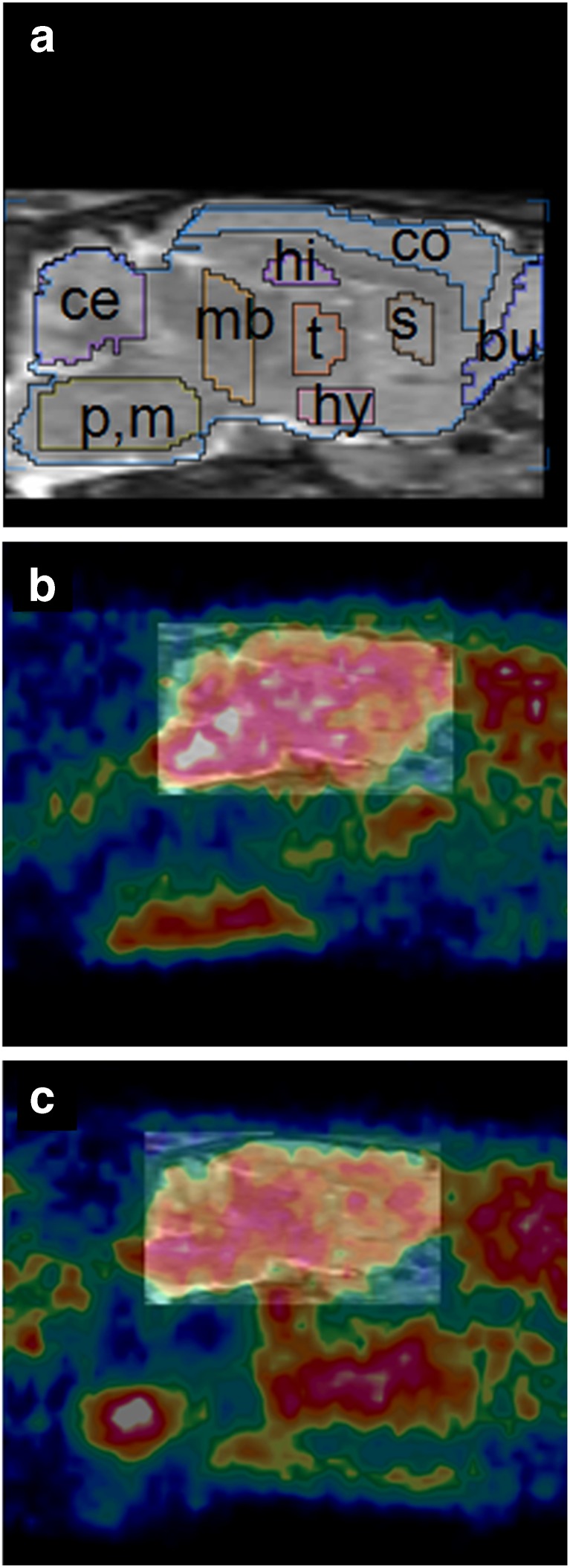
Images of rat brain (sagittal views, animals anesthetized with isoflurane). **a** MRI template. Brain regions are indicated by the following abbreviations: *bu* bulbus olfactorius, *ce* cerebellum, *co* cortex, *hi* hippocampus, *hy* hypothalamus, *mb* midbrain, *p*,*m* pons and medulla, *s* striatum, and *t* thalamus. **b** MicroPET image of young rat (age 3 months) superimposed on the MRI template. **c** MicroPET image of old rat (age 18 months) superimposed on this template. Frames from 22 to 74 min were summed (since early frames may contain a significant contribution of radioactivity in the blood pool), and a plane was chosen about 1.6 mm from the midline. Note the high uptake in the pons and medulla of the young rat whereas the distribution of radioactivity in the brain of the old rat is more uniform.

In the brain of young rats (ages 1.5 and 3 months), a characteristic regional pattern of tracer uptake was seen with the highest levels in the pons and medulla, followed by the midbrain, thalamus, and hypothalamus. Lower levels of radioactivity were noticed in the cortex, striatum, hippocampus, bulbus, amygdala, and cerebellum. In the brains of old rats (ages 18, 24, and 32 months), regional differences in tracer uptake were less obvious (Fig. 1).

### Radioactive Metabolites

**Fig. 2:**
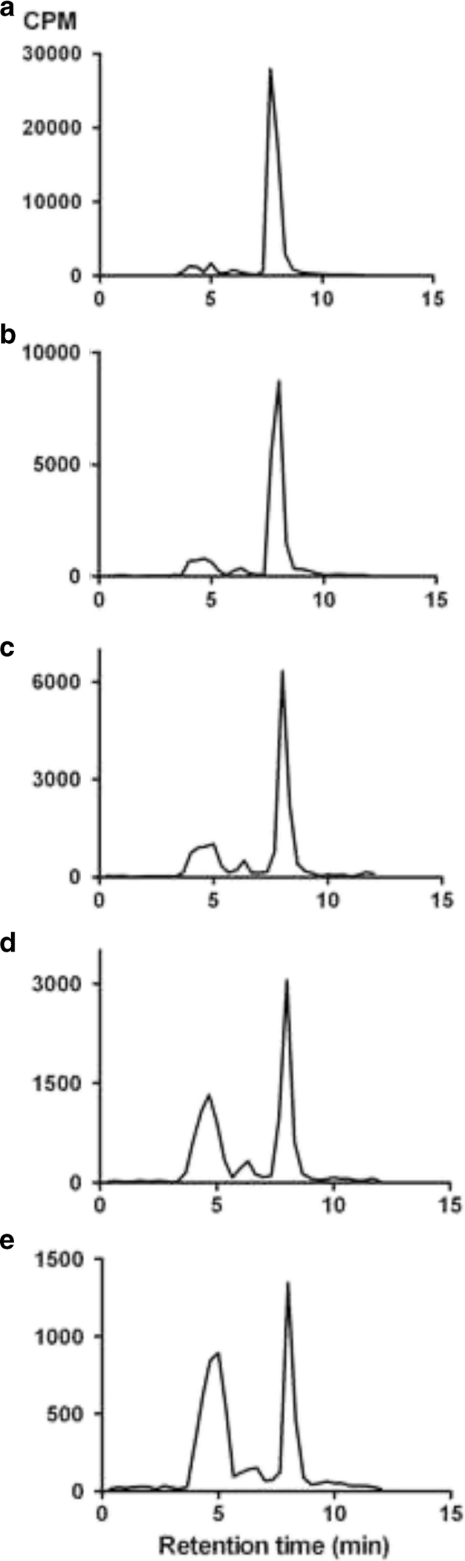
Radiochromatograms of rat plasma. Samples were drawn at different intervals after intravenous injection of [^11^C]SA4503: 5 min (**a**), 10 min (**b**), 20 min (**c**), 40 min (**d**), and 60 min (**e**). Eluate radioactivity (in CPM) is plotted against retention time on the HPLC column. The radioactive peak with 8-min retention time is the parent compound; radioactive species eluting between 4 and 6 min are metabolites.

In plasma of both young and old rats, a time-dependent formation of two radioactive metabolites was observed. These metabolites were hydrophilic and eluted at shorter retention times than the parent compound in reversed-phase HPLC (Fig. 2). Lipophilic radioactive species were not detected.

### Tracer Kinetics

**Fig. 3:**
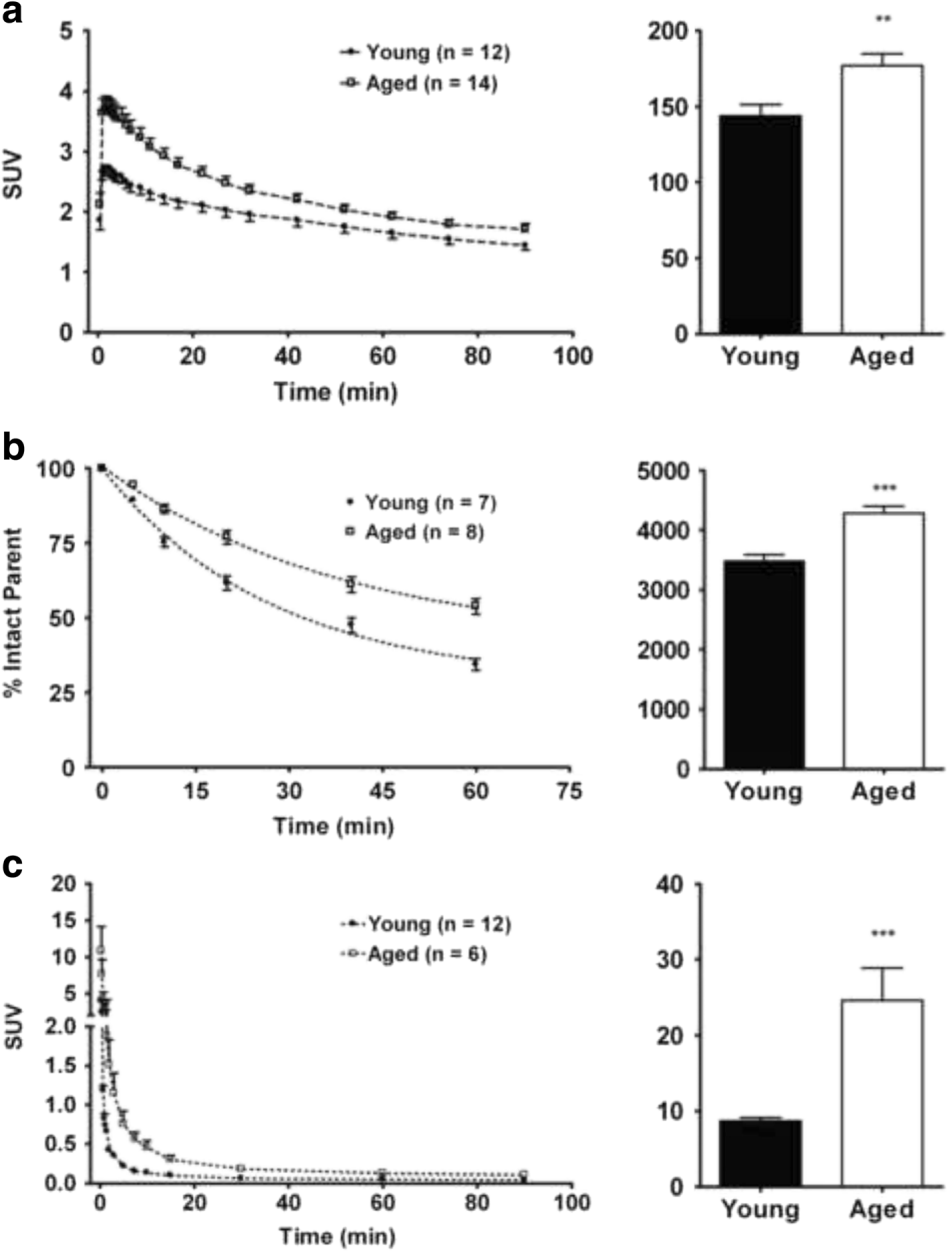
Tracer kinetics in brain and plasma of young and aged rats: whole brain TACs and AUCs (**a**), fraction of plasma radioactivity representing intact parent tracer as a function of time after injection and area under this curve (**b**), and metabolite-corrected TACs and AUCs in plasma (**c**). All data are plotted as mean ± SEM. ** indicates *P* < 0.01 and *** *P* < 0.001.

Tracer uptake in the brain was rapid, a peak at <5 min being followed by a slow washout (Fig. 3a). The area under the curve (AUC) for the whole brain was significantly greater at age 18 to 32 than age 1.5 to 3 months (*P* < 0.01). Metabolism of [^11^C]SA4503 was affected by aging. While only 34 % of plasma radioactivity at 60 min represented intact parent tracer in young rats, this fraction increased to 54 % in aged rats (Fig. 3b). The area under the parent fraction curve was significantly higher (*P* < 0.001) in aged rats.

Tracer clearance was rapid in both the age groups. Metabolite-corrected time-activity curves (TACs) of plasma radioactivity were best fitted by a tri-exponential function in the young and a bi-exponential function in the aged group. The AUC for aged animals was significantly higher (*P* < 0.001) than for young rats (Fig. 3c).

### Kinetic Analysis

**Fig. 4:**
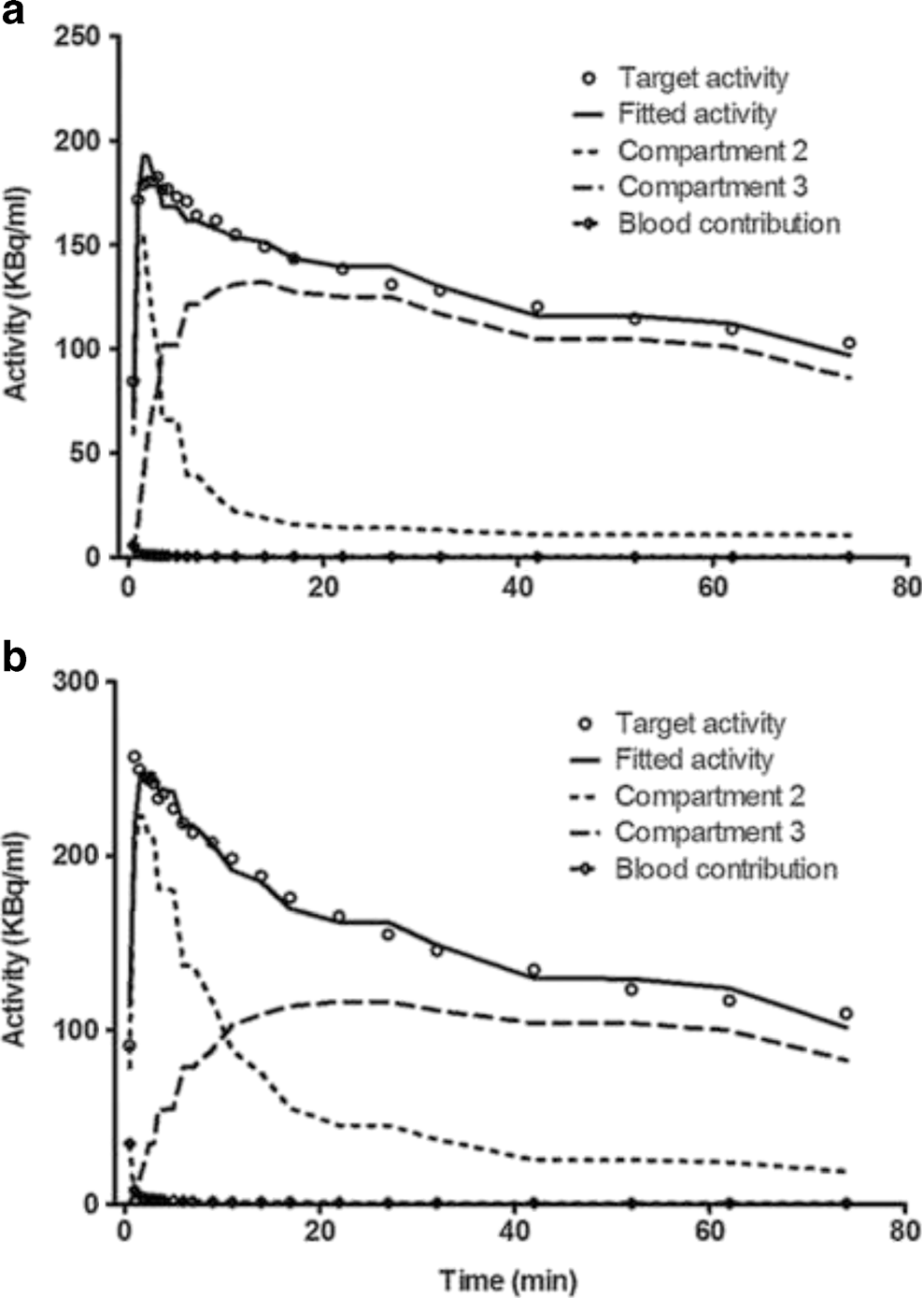
Kinetics of ^11^C]SA4503 in rat brain and the corresponding 2-TCM fits in young (**a**) and aged animal (**b**).

**Fig. 5:**
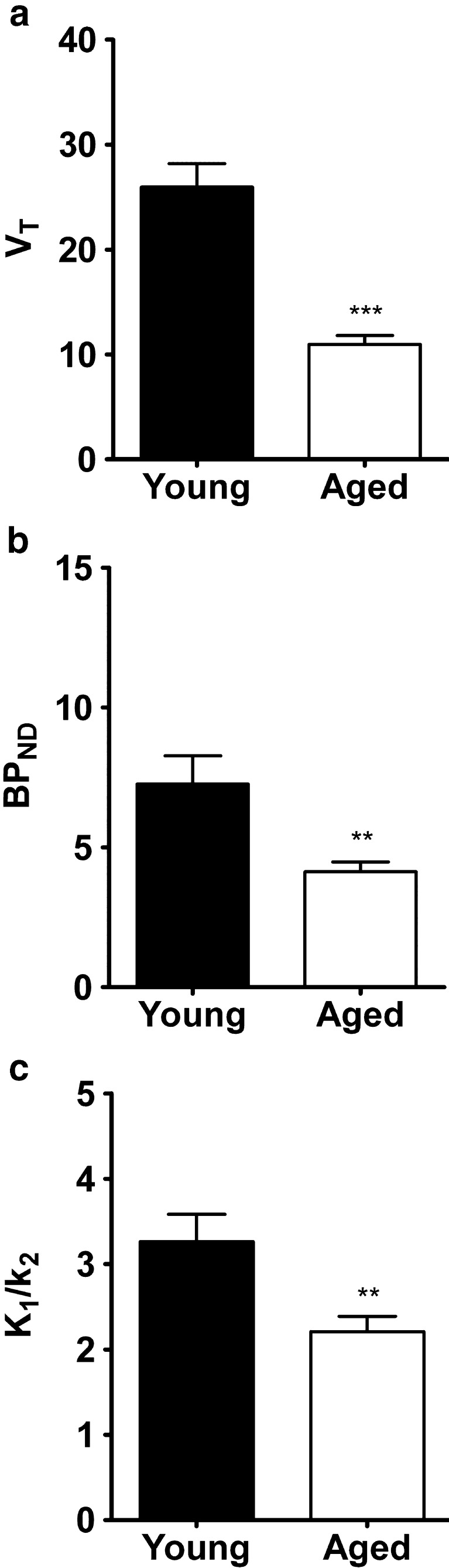
Data from a whole brain 2-TCM fit in young and aged rats. Plotted are *V*_T_ (**a**), *BP*_ND_ (**b**), and *K*_1_/*k*_2_ (**c**). **P < 0.01, ***P < 0.001.

A 2-TCM was fitted to TACs of ROIs drawn around the whole brain, using metabolite-corrected plasma radioactivity from arterial blood samples as input function. Representative model fits to PET data of a young and an aged rat are shown in Fig. 4. The fit indicated that tracer *V*_T_ was significantly reduced (*P* < 0.001) with aging (Fig. 5a). The fit parameters suggested that this reduction was due both to a lower *BP*_ND_ (*P* < 0.01) and a lower *K*_1_/k_2_ (*P* < 0.01) of the tracer in aged rats (Fig. 5b, c). *V*_T_ values calculated from a 2-TCM fit or by Logan graphical analysis were strongly correlated (*r*^2^ = 0.99, *P* < 0.0001), Logan analysis leading to a slight underestimation of about 5 % (see supplementary data).

**Fig. 6:**
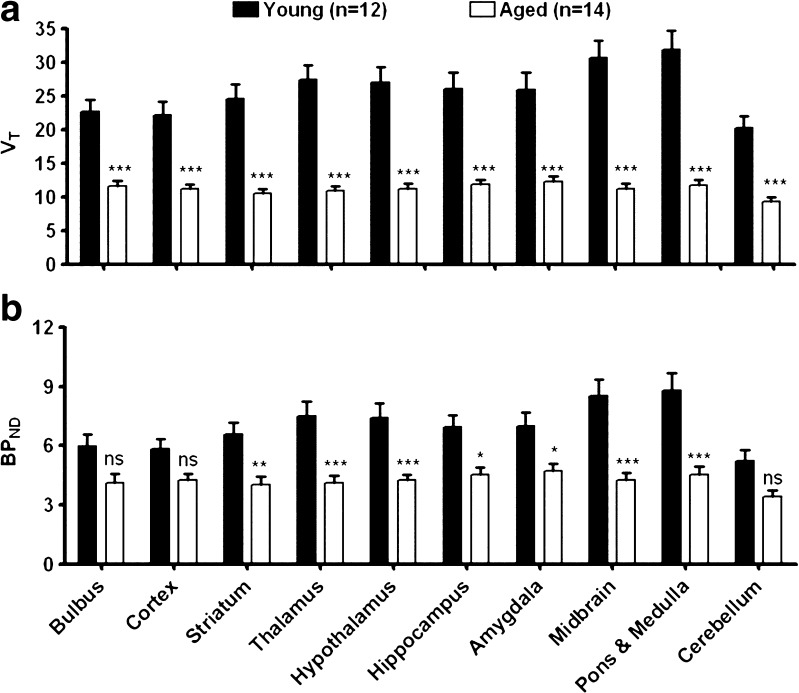
Data from 2-TCM fits in brain regions of young and aged rats. Plotted are *V*_T_ (**a**) and *BP*_ND_ (**b**). *P < 0.05, **P < 0.01, ***P < 0.001, *ns* not significant.

For analysis of tracer uptake in brain regions, a 2-TCM was fitted to TACs of ROIs derived from the MRI template in fused PET–MRI images. *K*_1_/*k*_2_ was fixed to the value estimated for the whole brain in each individual rat. Fixing of this ratio improves the fit particularly in small regions and reduces variability in the fit parameters. Tracer *V*_T_ in all the brain regions was significantly lower in the aged group (Fig. 6a). Tracer binding potential (*BP*_ND_) was also reduced by aging in most areas of the brain, but statistical significance was not reached in the bulbus, cerebral cortex, and cerebellum (Fig. 6b).

### Biodistribution

Biodistribution data for the brain and peripheral organs are listed in Table 2. At an interval of 90 min after tracer injection, aged rats showed a significantly lower SUV in the brain regions other than the cerebellum or cortex, and a significantly higher SUV in the spleen, submandibular gland, and urine. However, since plasma levels of radioactivity were different in aged and young rats, tissue-to-plasma ratios of radioactivity were a better measure of changes in organ uptake. Tissue-to-plasma ratios in the cerebral cortex were not significantly affected by aging, but ratios in the cerebellum and rest of the brain were significantly reduced in aged rats. Among peripheral tissues, the kidney and liver demonstrated a significant (*P* < 0.001) reduction of tissue-to-plasma ratio with aging.

**Table 2:** Biodistribution of [^11^C]SA4503 (90 min after injection)

	SUV			T/P ratio		
Tissue	Young (*n* = 16)	Aged (*n* = 13)	*P* value	Young (*n* = 16)	Aged (*n* = 13)	*P* value
Cerebellum	1.31 ± 0.09	1.33 ± 0.10	ns	12.70 ± 1.37	8.23 ± 1.00	<0.05
Cerebral cortex	1.30 ± 0.08	1.56 ± 0.11	ns	12.43 ± 1.06	9.66 ± 1.11	ns
Rest brain	1.75 ± 0.10	1.36 ± 0.09	<0.05	16.79 ± 1.52	8.45 ± 0.98	<0.001
Adipose tissue	0.47 ± 0.06	0.28 ± 0.06	ns	4.40 ± 0.56	1.66 ± 0.36	ns
Bladder	1.30 ± 0.20	2.34 ± 0.73	ns	13.04 ± 2.63	14.67 ± 5.14	ns
Bone	0.46 ± 0.05	0.43 ± 0.05	ns	4.19 ± 0.49	2.58 ± 0.31	ns
Bone marrow	2.29 ± 0.91	3.15 ± 0.73	ns	20.97 ± 8.38	17.67 ± 3.57	ns
Heart	0.36 ± 0.03	0.65 ± 0.04	ns	3.51 ± 0.44	3.92 ± 0.39	ns
Large intestine	1.91 ± 0.10	2.20 ± 0.16	ns	18.17 ± 1.53	13.51 ± 1.55	ns
Small intestine	2.29 ± 0.22	3.18 ± 0.26	ns	22.08 ± 2.96	19.49 ± 2.26	ns
Kidney	4.40 ± 0.23	3.50 ± 0.21	ns	42.00 ± 3.47	21.30 ± 1.89	<0.001
Liver	9.30 ± 0.39	10.78 ± 0.47	ns	87.37 ± 5.45	65.30 ± 5.37	<0.001
Lung	2.07 ± 0.11	2.24 ± 0.16	ns	19.69 ± 1.69	13.61 ± 1.38	ns
Muscle	0.17 ± 0.02	0.35 ± 0.06	ns	1.76 ± 0.27	2.13 ± 0.43	ns
Pancreas	5.18 ± 0.51	5.63 ± 0.59	ns	48.46 ± 5.86	34.75 ± 4.99	ns
Plasma	0.11 ± 0.01	0.17 ± 0.01	ns	1.00	1.00	ns
Red blood cells	0.06 ± 0.01	0.09 ± 0.01	ns	0.63 ± 0.07	0.50 ± 0.06	ns
Spleen	2.47 ± 0.17	4.45 ± 0.30	<0.01	23.52 ± 2.11	27.28 ± 2.81	ns
Submandibular gland	3.29 ± 0.25	5.98 ± 0.59	<0.001	30.82 ± 2.72	36.20 ± 4.58	ns
Urine	1.58 ± 0.28	3.32 ± 1.22	<0.05	14.51 ± 2.67	21.33 ± 8.50	ns

## Discussion

The regional distribution of radioactivity which we observed in the brain of young rats (high in the pons, medulla, midbrain, and hypothalamus; lower in the caudate–putamen and cerebellum, see Fig. 1) corresponds to published data for regional sigma-1 receptor expression in such animals, based on autoradiography [1], *in vitro* binding assays [33], and immunocytochemical techniques. [4] This regional pattern differs significantly from the distribution of sigma-2 receptors which predominate in other brain areas [2, 34]. The reduced regional variability of radioactivity in the aged brains suggests that the impact of aging on [^11^C]SA4503 uptake is region-specific, some areas showing greater changes than others, resulting in a more uniform distribution of radioactivity in the brain of aged rats.

In a previous study with [^11^C]SA4503, radioactivity in the rat brain was shown to represent only intact parent compound [35]. In the present study, we observed both in the aged and in young rats two hydrophilic radioactive metabolites in blood plasma which are not expected to enter the brain (Fig. 2). Lipophilic metabolites were not detected.

Standardized uptake values of radioactivity in the brain, and areas under the brain and plasma time-activity curves were significantly higher in the aged animals than in the young group (Fig. 3a, c). Higher brain and plasma levels in old animals could be related to the fact that aged rats weigh more than young ones and SUVs tend to positively correlate with animal weight. [36] However, the injected PET tracer was metabolized less rapidly in aged rats, resulting in a higher fraction of plasma radioactivity representing intact parent tracer (Fig. 3b). Injected radioactivity was also less rapidly cleared in old rats (Fig. 3c). The altered shape of the plasma curve (bi-exponential in aged versus tri-exponential in young rats), the slower kinetics of radioactivity in plasma, and the reduced tissue-to-plasma ratios of radioactivity in the kidney and liver of aged rats (Table 2) suggest that aged animals have reduced liver and kidney function. Since the injected probe was less rapidly metabolized and cleared, more tracer remained in plasma and was available for delivery to the brain.

Tracer levels in the circulation should be taken into account when groups are compared. Distribution volumes or binding potentials of the radioligand estimated by graphical analysis of PET data or by tracer-kinetic modeling can be used for this purpose [37]. Tissue-to-plasma ratios of radioactivity determined at a long interval after tracer injection could also be used as an estimate of receptor binding.

Tracer distribution volume and binding potential in the whole brain, calculated from a 2-tissue compartment model fit (Fig. 4), were reduced after aging (Fig. 5). When tracer binding potential was calculated for individual brain regions, some areas of the brain appeared to be little affected whereas other areas showed significant reductions. The greatest decreases were noted in the thalamus, hypothalamus, midbrain, pons, and medulla (Fig. 6). The fit data indicate that the more uniform distribution of radioactivity which we observed in the aged brain (Fig. 1) is due to substantial age-related losses of tracer binding in some areas (particularly pons/brainstem, medulla, midbrain, thalamus, and hypothalamus) whereas losses in other areas (particularly cortex) are smaller and statistically non-significant. Tissue-to-plasma ratios of radioactivity determined 90 min after tracer injection indicated that uptake in the cerebral cortex was not significantly affected by aging whereas uptake in the cerebellum and the rest of the brain was significantly reduced (Table 2). Thus, our data support a loss or down-regulation of sigma-1 receptors in most brain areas with aging, particularly in the pons, medulla, midbrain, thalamus, and hypothalamus, whereas sigma-1 receptors in the cerebral cortex appear to be rather well preserved.

Besides decreases of distribution volume and binding potential, the 2-TCM fit indicated a decrease of *K*_1_/*k*_2_, which suggests a decrease of tracer entry into the brain. In an American study on Sprague–Dawley rats (which are closely related to our own Wistar Hannover strain), a 25–33 % decline of regional cerebrovascular permeability surface area product was detected when animals aged from 6 to 24 months [38]. This decline corresponds closely to our observed decrease of *K*_1_/*k*_2_.

The strong correlation which we observed between estimations of *V*_T_ by 2-TCM fit and Logan plot suggests that Logan graphical analysis can be used as a robust method to quantify [^11^C]SA4503 uptake in the rodent brain, as we had noted previously [28, 29, 39].

Although our study is the first report of sigma-1 receptor imaging in rats of different ages, the impact of aging on sigma receptor expression in the rodent brain has been examined previously using *in vitro* assays. Unfortunately, most previous studies employed non-subtype-selective sigma ligands ([^3^H]-1,3-ditolylguanidine, [^3^H]haloperidol), so that measured receptor densities corresponded to the sum of the sigma-1 and sigma-2 receptor populations and cannot be compared to the present results with [^11^C]SA4503 [20, 21, 24]. A decline of sigma-1 receptor expression between birth and adult age (i.e., 50 days) was detected in the whole brain homogenates of Sprague–Dawley rats [22]. Using [^3^H]SA4503 as radioligand, a 4.5-fold increase of sigma-1 receptor density (B_max_) was observed in the whole brain of Fisher-344 rats between ages 1.5 and 24 months, but since the affinity of the receptor for the radioligand declined 3.8-fold during the same period, the binding potential (B_max_/K_d_) was only slightly increased [23]. Using [^3^H]-(+)-pentazocine in Fisher-344 rats, smaller (1.5-fold) increases of sigma-1 receptor density were noticed with corresponding (1.4-fold) decreases of ligand affinity and no significant change of binding potential [23]. Results for individual brain regions were not reported.

Reductions of sigma-1 receptor binding in the midbrain, pons, and medulla could be related to motor problems in aged animals, since sigma-1 receptors in these brain areas are primarily located on motor neurons and sigma-1 receptor knockout results in a reduced ability of mice to remain on the rotarod [40]. Pharmacological studies in isolated tissue have also suggested that brainstem sigma-1 receptors are involved in the regulation of motor responses [41]. Reduced sigma-1 receptor binding in the hypothalamus may be associated with impaired responses of aged animals to stress, since hypothalamic sigma-1 receptors are required for stimulation of brain-derived neurotrophic factor (BDNF) expression and the hypothalamus plays an important role in regulating the activity of the hypothalamus-pituitary-adrenal (HPA) axis [42].

## Conclusions

Our microPET study provides evidence for changes of sigma-1 receptor binding in the aging rat brain which are regionally different. Binding potential of the agonist tracer ^11^C]SA4503 was reduced in the (hypo) thalamus, midbrain, pons, and medulla, but rather well preserved in the cortex.

## Electronic Supplementary Material

Below is the link to the electronic supplementary material.

ESM 1 (PDF 241 kb)
