# Supplementary material for: Sigma-1 Agonist Binding in the Aging Rat Brain: a MicroPET Study with [11C]SA4503
Source: Mol Imaging Biol. 2015 Dec 4;18:588–97. doi: 10.1007/s11307-015-0917-6 (PMC4927617; doi:10.1007/s11307-015-0917-6)
Supplement: Supplementary file 1 — (PDF 241 kb) [file 11307_2015_917_MOESM1_ESM.pdf]

Electronic Supplementary Material

Sigma-1 Agonist Binding in the Aging Rat Brain: A MicroPET Study with [^{11}C]SA4503

Journal: Molecular Imaging and Biology

Nisha K. Ramakrishnan[1,2], Anniek K.D. Visser[1], Anna A. Rybczynska[1], Csaba J. Nyakas[3,4], Paul G.M. Luiten[3,4], Chantal Kwizera[1], Jurgen W.A. Sijbesma[1], Philip H. Elsinga[1], Kiichi Ishiwata[5], Rudi A.J.O. Dierckx[1], Aren van Waarde[1]

[1] University of Groningen, University Medical Center Groningen, Department of Nuclear Medicine and Molecular Imaging, Hanzeplein 1, 9713 GZ Groningen, The Netherlands

[2] Current address: King's College London, Division of Imaging Sciences and Biomedical Engineering, Strand, London WC2R 2LS, United Kingdom

[3] University of Groningen, Research Group of Molecular Neurobiology, Nijenborgh 7, 9747 AG Groningen, The Netherlands

[4] Department of Morphology and Physiology, Semmelweis University, 17 Vas, H-1088 Budapest, Hungary

[5] Tokyo Metropolitan Institute of Gerontology, Research Team for Neuroimaging, 35-2 Sakae-cho, Itabashi-ku, Tokyo 173-0015, Japan

Corresponding author: Aren van Waarde, e-mail: a.van.waarde@umcg.nl, Telephone: +31-50-3613215, Telefax: +31-50-361168

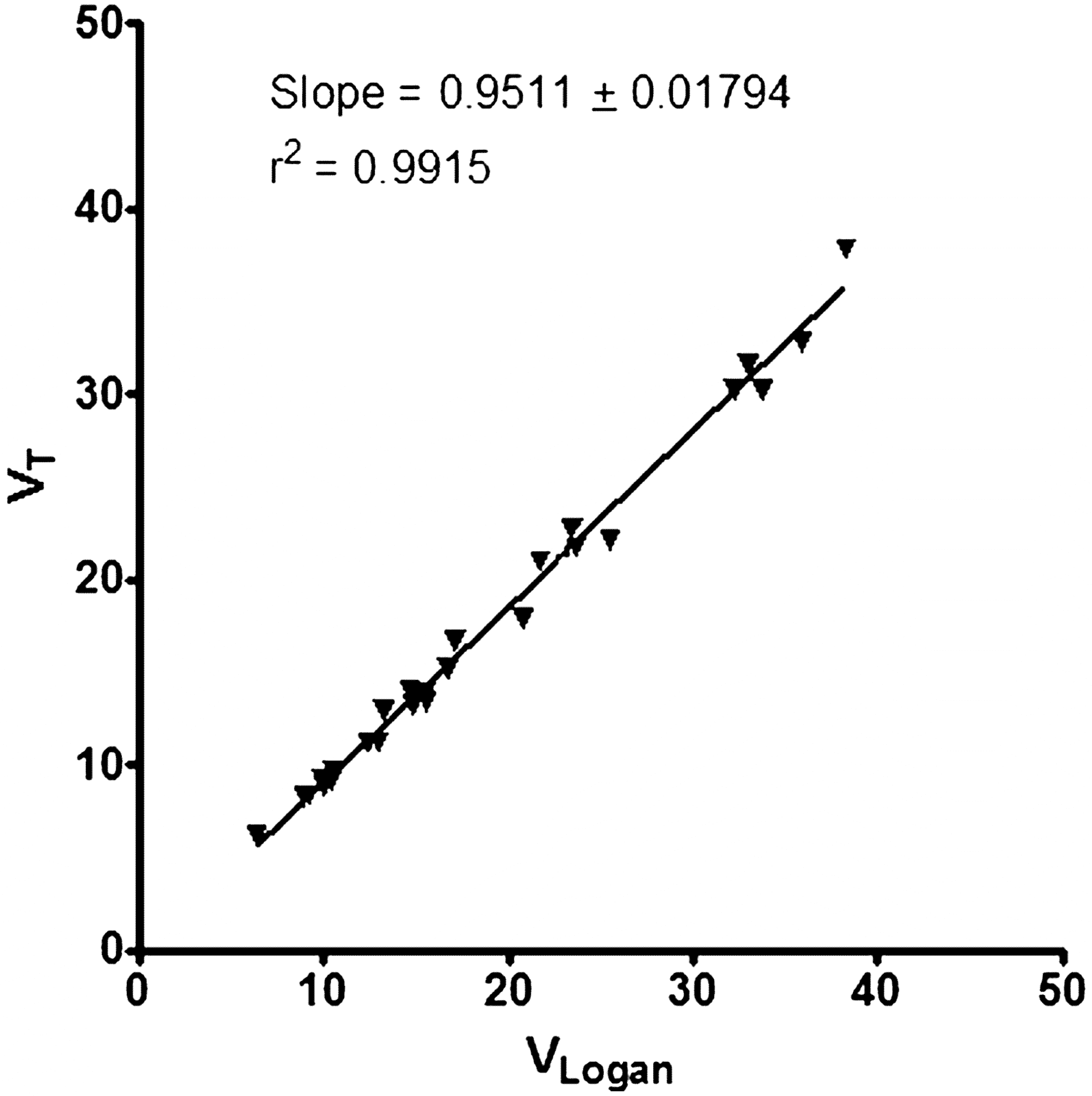

Supplemental Figure 1: V_T values calculated from a 2-TCM fit or by Logan graphical analysis were strongly correlated (r^2 = 0.99, P < 0.0001), Logan analysis leading to a slight underestimation of about 5% (see supplementary data).
